# *Hmox1* Upregulation Is a Mutual Marker in Human Tumor Cells Exposed to Physical Plasma-Derived Oxidants

**DOI:** 10.3390/antiox7110151

**Published:** 2018-10-27

**Authors:** Sander Bekeschus, Eric Freund, Kristian Wende, Rajesh Kumar Gandhirajan, Anke Schmidt

**Affiliations:** ZIK plasmatis, Leibniz Institute for Plasma Science and Technology (INP), Felix-Hausdorff-Str. 2, 17489 Greifswald, Germany; eric.freund@inp-greifswald.de (E.F.); kristian.wende@inp-greifswald.de (K.W.); rajesh.gandhirajan@inp-greifswald.de (R.K.G.); anke.schmidt@inp-greifswald.de (A.S.)

**Keywords:** heme oxygenase 1, kINPen, plasma medicine, reactive oxygen and nitrogen species, RNS, ROS

## Abstract

Increasing numbers of cancer deaths worldwide demand for new treatment avenues. Cold physical plasma is a partially ionized gas expelling a variety of reactive oxygen and nitrogen species, which can be harnesses therapeutically. Plasmas and plasma-treated liquids have antitumor properties in vitro and in vivo. Yet, global response signatures to plasma treatment have not yet been identified. To this end, we screened eight human cancer cell lines to investigate effects of low-dose, tumor-static plasma-treated medium (PTM) on cellular activity, immune-modulatory properties, and transcriptional levels of 22 redox-related genes. With PTM, a moderate reduction of metabolic activity and modest modulation of chemokine/cytokine pattern and markers of immunogenic cell death was observed. Strikingly, the Nuclear factor (erythroid-derived 2)-like 2 (*nrf2*) target heme oxygenase 1 (*hmox1*) was upregulated in all cell lines 4 h post PTM-treatment. *nrf2* was not changed, but its baseline expression inversely and significantly correlated with *hmox1* expression after exposure to PTM. Besides awarding *hmox1* a central role with plasma-derived oxidants, we present a transcriptional redox map of 22 targets and chemokine/cytokine secretion map of 13 targets across eight different human tumor cell lines of four tumor entities at baseline activity that are useful for future studies in this field.

## 1. Introduction

Cancer is the second leading cause of death worldwide [1]. Hence, global research efforts aim at better understanding cancer pathology and the discovery of new therapeutic avenues to combat disease. While there is a plethora of small molecules and biologicals being investigated for antitumor effects, research on a number of technological approaches supporting therapies or targeting cancer is carried as well. This includes studies using nanoparticles [2], photodynamic therapy [3], high hydrostatic pressure [4], acoustic treatment [5], hyperthermia [6], ionizing radiation combinational therapies [7], and cold-physical plasma-based approaches [8]. Cold physical plasma is a partially ionized gas that is generated at body temperature and composed of multiple effectors, such as electric fields, light radiation, ions and electrons, and reactive oxygen and nitrogen species (ROS/RNS) [9]. The later are hypothesized to be the main component responsible for antitumor effects observed with plasma treatment [10]. This includes the inactivation of tumor cells in vitro [11,12,13] and in vivo [14,15,16]. The use of plasma-treated liquids directly injected into tumors or used as lavage in peritoneal carcinomatosis has been motived in vivo as well [17,18,19]. Recently, first patients suffering from head and neck cancer benefited from plasma therapy in the palliative stage [20,21,22].

Despite many studies reporting on antitumor effects of cold physical plasma, molecular mechanism responsible for the effects observed are less investigated. However, common (bio-) markers that are associated with plasma treatment are missing. Along similar lines, there is an unmet need for the identification of markers for other types of therapies including ionizing radiation [23] and photodynamic therapy (PDT) [24]. Established markers would allow not only tracking, but also for quantifying the therapeutic efficacy of a given treatment.

Plasma treatment release ROS/RNS, and these are known to act in redox signaling pathways. A major pathway is that of the Nuclear factor (erythroid-derived 2)-like 2 (*nrf2*). *nrf2* signaling is important in many physiological and pathological conditions, including cancer [25]. Once released from Keap1 under oxidative stress conditions, *nrf2* translocates to the nucleus where it binds phase II enzymes of the antioxidant response. Next to NAD(P)H dehydrogenase [quinone] 1 (gene: *nqo1*; protein: Nqo1) [26], heme oxygenase 1 (gene: *hmox1*; protein: HO1) is among the *nrf2* target genes. It catalyzes heme degradation with biliverdin, iron ions, and carbon monoxide (CO) as by-product, and HO-1 has been associated with ROS/RNS-driven oxidative stress responses [27]. A large number of *nrf2*-independent and dependent proteins are enrolled in the redox signaling pathways, such as peroxiredoxins, glutaredoxins, and thioredoxin [28,29,30].

Given the importance of ROS/RNS in cancer biology and the prominent role of redox proteins and their subsequent signaling functions, we investigated the responses of eight human cancer lines to plasma-derived ROS/RNS. Specifically, the cellular activity, growth, cytokine/chemokine profile, quantitative expression of immune-relevant cell surface markers, and transcriptional levels of 22 redox-related proteins was investigated. While plasma-derived ROS/RNS were of modest toxicity, *hmox1* was identified as a common responder to that treatment in all eight human cancer cell lines. 

## 2. Materials and Methods 

### 2.1. Cell Culture

Eight human and referenced (reference number in parentheses) cell lines were used in this work, including SK-Mel 28 (SKM, human malignant melanoma, HTB-72), MNT-1 (MNT1, human malignant melanoma, CVCL_5624), Capan-1 (Capan1, human pancreatic adenocarcinoma, HTB-79), Panc-01 (Panc01, human pancreatic adenocarcinoma, CRL-1469), HT-29 (HT29, human colorectal adenocarcinoma, HTB-38), SW-480 (SW480, human colorectal adenocarcinoma, CCL-228), MCF-7 (MCF7, human mammary adenocarcinoma, HTB-22), and MDA-MB-231 (MDA, human mammary adenocarcinoma, HTB-26). Cells were cultured in Roswell Park Memorial medium (RPMI1640) or Dulbecco’s Modified Eagle Medium (DMEM), each supplemented with 10% fetal bovine serum, 2% penicillin/streptomycin, and 1% L-glutamine (all Sigma, Taufkirchen, Germany). For seeding cells in 60 mm dishes (Sarstedt, Nuembrecht, Germany) at 1 × 10^6^ per dish for transcription experiments or 96-well plates (Eppendorf, Hamburg, Germany) at 1 × 10^4^ per well for all other experiments, accurate viable cell counts were retrieved using flow cytometry (attune; Applied Biosystems; Foster City, CA, USA) and 4′,6-Diamidin-2-phenylindol (DAPI; Sigma, Taufkirchen, Germany) to exclude dead cells. After seeding, cells were allowed to attach overnight prior to experimentation. The 96-well plates were equipped with a rim that was filled with phosphate-buffered saline (PBS; Pan Biotech, Aidenbach, Germany) to avoid evaporation in the outer wells. All the cultivations were done at 37 °C, 95% humidity, and 5% CO_2_ in a cell culture incubator (Binder, Tuttlingen, Germany).

### 2.2. Plasma-Treated Medium (PTM)

Plasma-treated medium (PTM) was prepared by exposing 50 mL of medium in a 250 mL glass beaker to the plasma of an atmospheric pressure argon plasma jet (kINPen; neoplas, Greifswald, Germany) for 60 min. Control medium was prepared for exposing 50 mL to argon gas only for 60 min. The plasma jet was operated with five standard liters per minute of argon (Air Liquide, Berlin, Germany), at a frequency of 1 Mhz, and a total power of less than 3.5 W in the handheld device. After plasma or argon gas treatment of the liquids, a pre-determined amount of double-distilled water was added to the liquids to compensate for evaporation. Media were then stored at −20 °C in aliquots prior to use within seven days. The feasibility of this approach has been documented previously [31,32,33]. For treatment of cells in 60 mm dishes, overnight culture medium was removed, cells were washed with PBS, and 5 mL of PTM or argon gas-treated medium were added to dishes before returning them to the incubator for another 4 h. For the treatment of cells in 96-well plates, overnight culture medium was removed, cells were washed with PBS, and 50 µL of PTM or argon-gas treated medium were added to each well (in quadruplicate per condition).

### 2.3. Live Cell Imaging

For live cell imaging, the 96-well plate was placed in a high content imaging device (Operetta CLS, PerkinElmer, Hamburg, Germany) equipped with a temperature module (37 °C and 5% CO_2_). The outer rim of the Eppendorf 96-well plate protected the outer wells from excessive evaporation during the 4 h of time lapse imaging. Images were acquired with laser-based autofocus every 15 min with a 20× air objective (numerical aperture 0.4; Zeiss, Oberkochen, Germany) and a 4.7 megapixels scientific complementary metal-oxide-semiconductor camera (sCMOS). Image mode was digital phase contrast (DPC), a label-free visualization method of the cytosolic area of cells. A standardized de-focusing procedure and software algorithm generates contrast-rich cell areas. More than 20,000 images were acquired across all experiments. After flat-field correction, quantification of these images was carried out with *Harmony 4.8* analysis software (PerkinElmer, Hamburg, Germany).

### 2.4. Multiplex Chemokine/Cytokine Analysis

Thirteen different immune-relevant targets were investigated using LegendPlex (BioLegend, London, UK) multi-analyte assay, a bead-based sandwich immuno assay. These beads differ in size and fluorescence intensity and thereby allow for the separate quantification of 13 targets in parallel by flow cytometry. The specific chemokines and cytokines quantified were arginase, CC-chemokine ligand (CLL) 17, C-X-C motif chemokine ligand (CXCL) 1, CXCL10, interferon (IFN) γ, interleukin (IL)1β, IL6, IL8, IL10, IL12, tumor growth factor (TGF) β, tumor necrosis factor (TNF) α, and vascular endothelial growth factor (VEGF). The supernatants were taken before harvesting tumor cells for quantitative polymerase chain reaction (qPCR) experiments, allowing for conclusions from one dataset to the other. The experimental procedure was performed in accordance to the supplier’s instructions and the data from flow cytometry (using CytoFLEX S, Beckman-Coulter, Brea, CA, USA) were analyzed using LegendPlex 8.0 analysis software (VigeneTech, Carlisle, MA, USA). Absolute concentrations (in pg/mL) were calculated from an asymmetric sigmoidal model from each target’s standard curve.

### 2.5. Cell Surface Marker Analysis

To determine the expression of immunologically relevant surface molecules (Table 1), tumor cells were detached 4 h after initial exposure to untreated control medium or PTM with accutase (BioLegend, London, UK). After washing with PBS, cells were incubated with DAPI as well as fluorescently-labeled antibodies targeted against cluster of differentiation (CD) 47 PerCP-Cy5.5 (BioLegend, London, UK), calreticulin (CRT) Alexa Fluor (AF) 647 (Novus Biologicals, city, Germany), human leukocyte antigen (HLA)-ABC phycoerythrin (PE)-Cy7 (Becton-Dickinson, Heidelberg, Germany), heat-shock protein (HSP) 70 AF488 (Abcam, Cambridge, UK), and HSP90 AF700 (Novus Biologicals, Cambridge, MA, USA). After incubation for 15 min in the dark, cells were washed, resuspended, and acquired with a CytoFLEX S flow cytometer (Beckman-Coulter, Brea, CA, USA). Quantification of the mean fluorescent intensities (MFI) was carried out using *Kaluza 2.1* analysis software (Beckman-Coulter, Brea, CA, USA). Fluorescence spillover was compensated while using single- and un-stained cells.

### 2.6. Quantitative Polymerase Chain Reaction (qPCR)

After 4 h of incubation with control or PTM, the cells were scratched off the dishes and transferred into 1.5 mL tubes (Eppendorf, Hamburg, Germany). After pelleting and suspending in lysis buffer, RNA isolation was performed according to the protocol of RNA isolation kit (RNA Mini Kit; Bio&SELL, Feucht, Germany). The RNA concentration of each sample was measured by using the NanoDrop 2000C (Thermo, Waltham, MA, USA) device was aliquoted into micro-tubes for further experiments. For quantitative polymerase chain reaction (qPCR), 1µg of RNA was synthesized into cDNA, according to the manufacturer’s instructions (ThermoFisher, Waltham, MA, USA) using a thermocycler (Biometra, Goettingen, Germany). qPCR was performed in white 96-well V-bottom plates with Sybr Green (BioRad, Munich, Germany) labeled targets over 50-cycles using a Light Cycler 480 machine (Roche, Mannheim, Germany). Fold changes in expression was calculated using the 2^−ΔΔ*C*t^ method, and normalized against glyceraldehyde 3-phosphate dehydrogenase (*gapdh*).

### 2.7. Statistical Analysis

Data are from 3–8 independent experiments performed with four technical replicates each. For normalization and calculation with raw data, Excel 2016 (Microsoft, Redmond, WA, USA) was utilized. Statistical analysis was performed with prism 7.05 (GraphPad software, San Diego, CA, USA). Bars show mean and standard error. Statistical comparison was performed either using paired *t*-test, or one-way or two-way analysis of variances (anova) to compare multiple groups. Levels of significance were indicated, as follows: *α* = 0.05 (*), *α* = 0.001 (**), *α* = 0.001 (***). 

## 3. Results

### 3.1. Plasma-Treated Medium Reduces the Metabolic Activity and Induces Swelling in Tumor Cells

**Figure 1:**
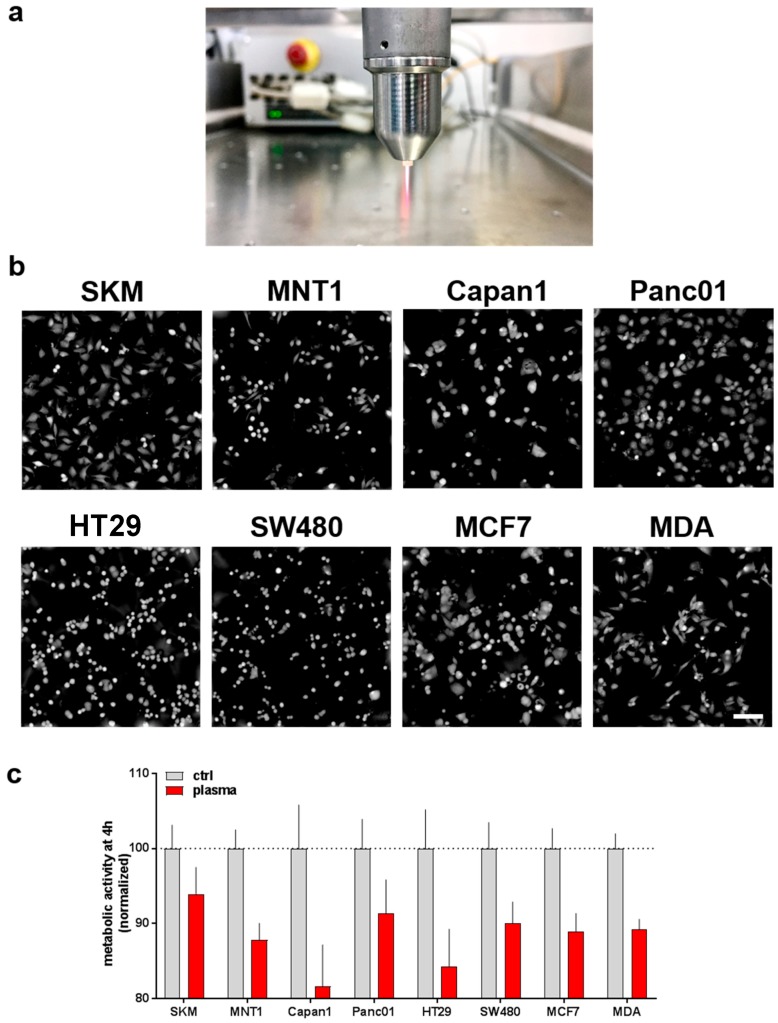
Exposure of initially-seeded 1 × 10^4^ cells of eight human tumor cell lines to plasma-treated medium led to a reduction in metabolic activity. (**a**) Representative image of the atmospheric pressure argon plasma jet (kINPen) utilized; (**b**) representative images in digital phase contrast (DPC) mode showing cytosolic fraction of all cell types investigated; (**c**) metabolic activity (as measured using resazurin) 4 h following exposure to control or PTM, data are normalized to that of control medium. Data are from 8 independent experiments with four technical replicates each. Scale bar = 100 µm. Red bars = plasma-treated medium (PTM); grey bars = untreated medium (control).

The kINPen plasma jet (Figure 1a) was used to deposit reactive oxygen and nitrogen species (ROS/RNS) into cell culture medium (plasma-treated medium, PTM). Control or PTM was given to eight different human tumor cells having intrinsic variation in morphology and growth pattern (Figure 1b). After four hours of incubation, the metabolic activity was assessed. A moderate but consistent decreases of metabolic activity was observed among all eight cell lines investigated (Figure 1c). 

**Figure 2:**
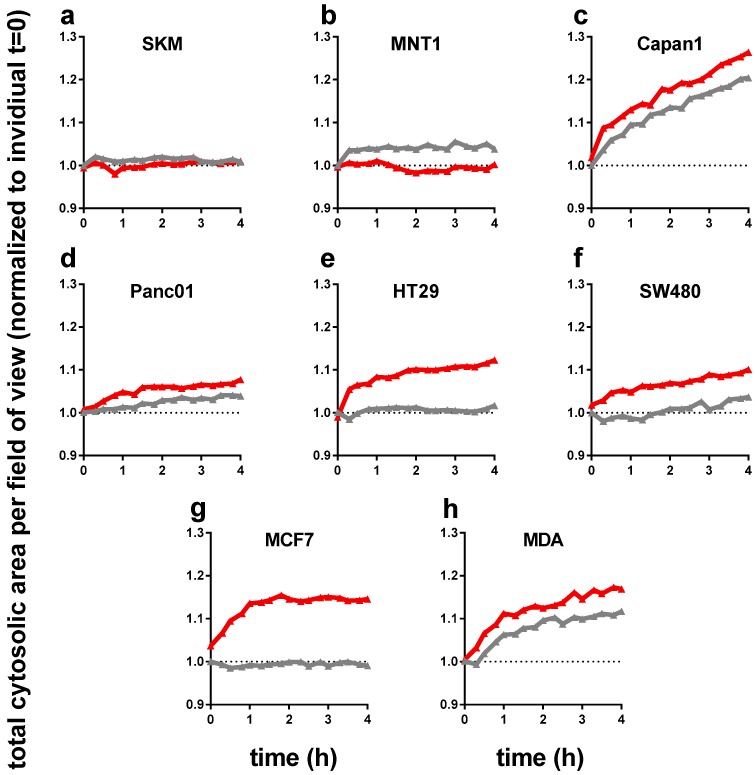
The total cytosolic cell growth area significantly increased with PTM (red) in six out of eight tumor cell lines (in 96-well plates with 1 × 10^4^ cells initially seeded). Time-dynamic data are from high-content microscopy in digital phase contrast mode (indicating cytosolic area, see materials and methods Section 2.3) and summarize data from over 20,000 single images. (**a**) SK-Mel 28, (**b**) MNT-1, (**c**) Capan 1, (**d**) Panc 01, (**e**) HT 29, (**f**) SW 480, (**g**) MCF7, and (**h**) MDA-MB-231; untreated medium control and PTM were normalized to each *t* = 0. Data are from three independent experiments with four technical replicates each. PTM = plasma-treated medium (red lines); control = untreated medium (grey lines).

Next, we performed live cell time lapse imaging of all eight cell lines for up to 4 h following incubation either with control or PTM. For each well, the total cellular cytosolic area (as measure of accumulated cellular spread of all cells identified per field of view) was quantified to normalize to t = 0 for each sample to monitor changes of time. Except melanoma cells (Figure 2a,b), all cell types showed a significant increase in total cytosolic area (Figure 2c–h). As some these changes were sudden within the first hour after treatment, we attribute this behavior to cellular swelling that is often associated with cellular repair and cycle arrest, which may be linked to the findings of reduced metabolic activity (Figure 1c). This effect was particular pronounced in HT29, SW480, and MCF7, and rather moderate in pancreatic tumor cell lines Capan1 and Panc01. Changes in the osmolality of PTM can be excluded as not all cell types exposed to PTM changed (e.g., SKM and MNT1) and some cell types increased their sum cytosolic area also in control medium (e.g., Capan1 and MDA).

### 3.2. Baseline and Regulated Transcriptional Level 22 Redox-Related Genes in Eight Human Cancer Cell Lines Following Exposure to Physical Plasma-Derived Oxidants

**Figure 3:**
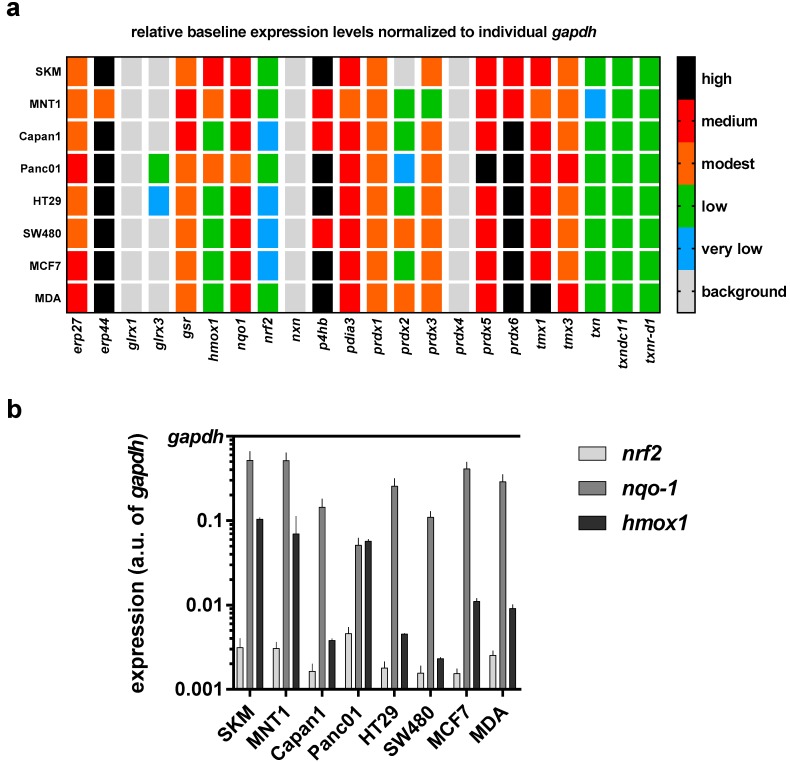
Heat map of transcriptional levels of 22 redox-related proteins in relation to cell line-specific levels of the house keeping gene *gapdh* as assessed via qPCR. (**a**) relative baseline values for all transcripts investigated in eight different human cell lines; (**b**) data from heat map represented for three targets known to respond to oxidative stress (*nrf2*, *nqo1*, and *hmox1*) for better individual comparison. Data are from three to four independent experiments.

In further experiments, we sought to profile in all eight cancer cell lines the transcriptional level of 22 redox-related genes that may be associated with the perception of ROS/RNS released by physical plasma into cell culture medium. To gain a better understanding of the differences among all cell lines, a mini redox-map was generated to assess the baseline expression levels of all targets investigated via qPCR (Figure 3a). Using *gapdh* levels as cell-specific normalization control, endoplasmic Reticulum protein 27.7 kDa (*erp27*), *erp4*, *nqo1*, protein disulfide-isomerase/beta-subunit of prolyl 4-hydroxylase (*p4hb*), protein disulfide isomerase family A member 3 (*pdia3*), *peroxiredoxin 5* (*prdx5*), and *prdx6*, as well as thioredoxin related transmembrane protein 1 (*tmx1*) and *tmx3* showed a medium to high expression in cells cultured in control medium. Transcripts of *glrx1*, *nxn*, and *prdx4* were not detected in any cell line. Moreover, the expression of *nrf2*, *nqo1*, and *hmox1* is shown in comparison to *gapdh* at higher resolution as these targets are known to be modulated upon oxidative stress (Figure 3b). A relatively low expression of *nrf2* in all eight cell lines is observed, whereas *nqo1* is mostly highly expressed. The biggest differences of expression between all cell lines investigated can be seen for *hmox1*. 

**Figure 4:**
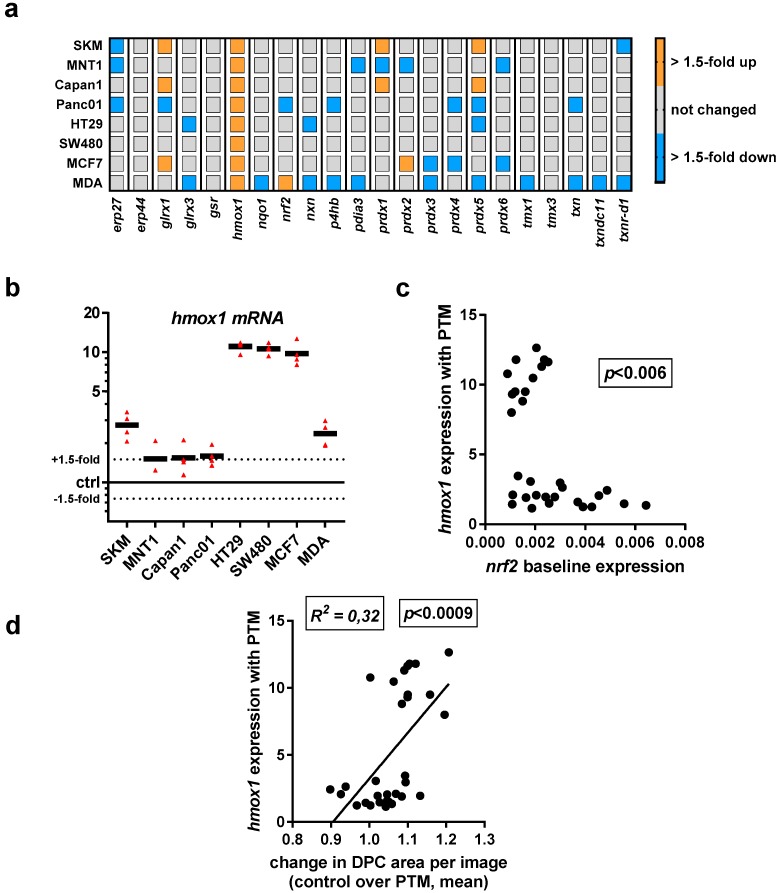
Exposure to PMT modulated transcriptional levels of several redox-related proteins with a consistent increase in *hmox1* transcripts across all eight human cancer cell lines investigated. Gene expression of redox-related enzymes, with upregulation of *glrx1* and *hmox1*, and a mixed response for peroxiredoxin genes, especially *prx1* and *prx5*. (**a**) qPCR of all target transcripts as fold change of PTM-treatment over medium control; (**b**) detailed expression levels of *hmox1* in all cell lines investigated; (**c**) relation between *hmox1* upregulation with PTM treatment and *nrf2* expression of cells incubated with control medium; (**d**) correlation of total cytosolic area determined as DPC area in high-content imaging experiments 4 h post plasma-treatment and expression of *hmox1*. Data are from three to four independent experiments. (**b**) each triangle and (**c**,**d**) each dot represents one biological replicate of one cell lines for the indicated markers.

Having investigated the baseline transcriptional expression levels of 22 redox-related proteins in eight human cancer cell lines, we investigated their up or downregulation following exposure to PTM at 4 h. We did not identify a consistent change with all except one target (*hmox1*) quantified across all cell lines used in this study (Figure 4a). A mixed response was observed for *glrx1*, *prdx1*, and *prdx5*. A more detailed analysis display of *hmox1* data revealed a pronounced upregulation, particularly with HT29, SW480, and MCF7 cells (Figure 4b). As *hmox1* is known to be a target of *nrf2*, correlation analysis was performed between baseline *nfr2* expression levels and *hmox1* expression levels induced with PTM. A significant inverse correlation was observed between both factors (Figure 4c). Especially for the three cell lines with high upregulation of *hmox1* following PTM exposure, a rather low *nrf2* baseline expression was observed. Interestingly, through quantification of the DPC signal after challenging the cells with ROS/RNS, we found a correlation between the expression of *hmox1* and the change in cytosolic area (Figure 4d). Hence, a high *hmox1* expression is characteristic, together with an increased cell area after PTM treatment.

### 3.3. Modest Changes in the Immunomodulatory Profile in Response to PTM in Eight Human Cancer Cell Lines 

**Figure 5:**
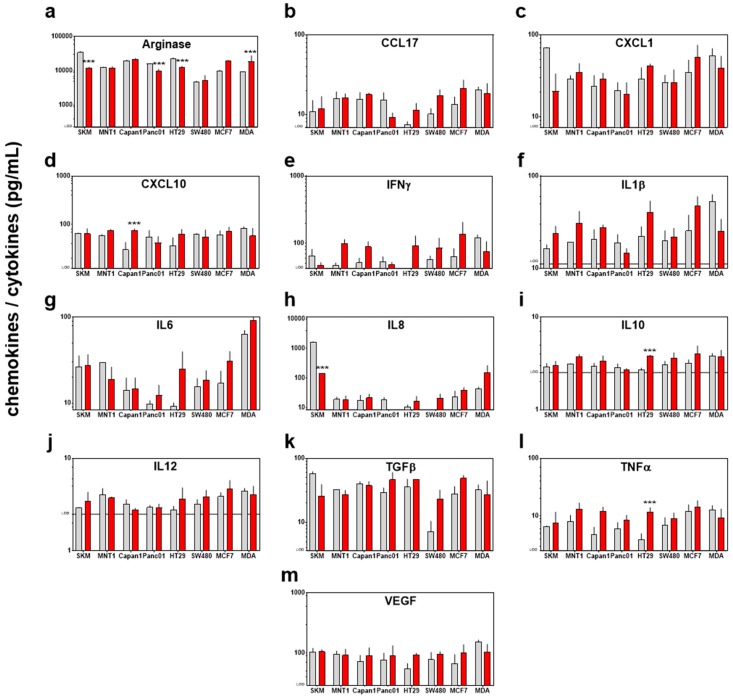
Exposure to PTM alters the chemokine/cytokine profile in supernatants of eight human cancer cell lines. Quantification of (**a**) arginase, (**b**) CCL17, (**c**) CXCL1, (**d**) CXCL10, (**e**) IFNγ, (**f**) IL1β, (**g**) IL6, (**h**) IL8, (**i**) IL10, (**j**) IL12, (**k**) TGFβ, (**l**) TNFα, and (**m**) VEGF; all concentrations are pg/mL; lines indicate the target-specific limit of detection (LOD). Data are pooled from eight independent experiments. Red bars = plasma-treated medium (PTM); grey bars = untreated medium (control).

Having analyzed the tumor-static effects of PTM with subsequent changes in the transcriptional profile of 22 redox-related proteins, we sought to investigate immunomodulatory effects of PTM treatment. An expression of *hmox1* is known to be tightly associated with inflammation [39]. Essential for such immune reaction is the secretion of inflammatory mediators. Therefore, we determined such soluble mediators in the cells supernatant and found that PTM induced a complex secretion profile of chemokines and cytokines in the different cell lines (Figure 5a–m). A significantly higher secretion of the pro-inflammatory mediators CXCL10 (in Capan1), arginase (in MDA), and TNFα (in HT29) was found, as well as a higher secretion of the anti-inflammatory interleukin 10 (in HT29) and the depletion of IL8 (in SKM) and arginase (in SKM, Panc01, HT29, and MDA). However, to evaluate the immunogenic effect of the secretion of different factors a more complex model, including different cell types is needed and our observations suggest a mixed response.

**Figure 6:**
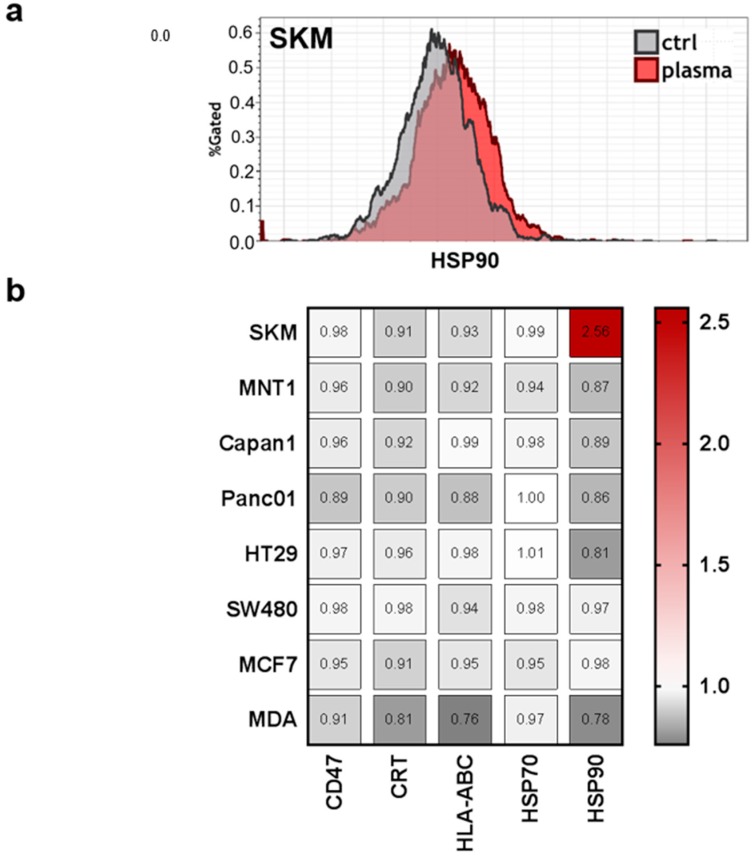
Expression of immune-response relevant surface markers remains predominantly unchanged after plasma treatment. Cells were harvested, stained with anti-cluster of differentiation 47 (CD47, PerCP-Cy5.5), anti-calreticulin (CRT, AF647), anti-human leucocyte antigen ABC (HLA-ABC, PE-Cy), anti-heat-shock protein 70 (HSP70, AF488), and anti-heat-shock protein 90 (HSP90, AF700) and were measured by flow-cytometry. (**a**) Representative overlay of expression of HSP90 in SKM28 cells; (**b**) quantification of mean fluorescence intensities (MFIs) on viable cells normalized on each’s cell lines untreated medium control.

Moreover, previous studies indicated that tumor-toxic concentrations of plasma-derived ROS/RNS are capable of inducing the immunogenic cancer cell death (ICD) [40]. This type of cell death promotes antitumor immune responses, and is characterized by release upregulation of a number of molecules on the cell membrane, such as calreticulin (CRT), major histocompatibility complex I (MHC class I; HLA-ABC), and heat-shock proteins (HSP) 70 and 90 [41]. This may be accompanied by a downregulation of CD47, which promotes the phagocytosis of tumor cells [34]. However, only SK-Mel 28 (SKM) cells showed a considerable increase of HSP90 on the membrane of living cells (Figure 6a). For all other surface molecules and cell lines, there were only minor changes that were observed in response to PTM at 4 h when normalized each respective control (Figure 6b). This may be due to the overall mild effect (short plasma treatment time) of the PTM. 

## 4. Discussion

In need of a common marker delineating the response of cancer cells to plasma-treated medium (PTM), we studied of growth behavior, immunogenic features, and the expression of 22 redox-related transcripts in eight human cancer cell lines. Besides presenting a small redox map in all cell types with regard to baseline redox-protein transcript expression and chemokine/cytokine profile, *hmox1* was identified as key response element in all cancer cell lines following exposure to PTM.

Angiogenesis is a key hallmark of cancer [42]. New blood vessel help support tumor growth by removing metabolic waste and carbon dioxide, and increasing nutrient supply. Induced by VEGF and *nrf2* as well as others, heme oxygenase 1 (HO1, *hmox1*) derived carbon monoxide (CO) possess not only proangiogenic effects, but also anti-inflammatory, antioxidant, and anti-apoptotic properties [43]. HO1 moreover detoxifies heme into ferrous iron, as the former would otherwise contribute to the cytotoxic fenton reaction generating hydroxyl radicals [44]. HO1 has therefore implicated as major target in cancer therapy although its expression is linked to both tumor progression and regress [45]. Oxidative stress is a known inducer of HO1 in human cells [46,47], underlining reactive oxygen and nitrogen species being a major bio-active component in physical plasma-treated liquids. Indeed, both human keratinocytes [48] and the human cell line THP-1 monocytes [49] upregulate *hmox1* in response to plasma-derived ROS/RNS.

While the identification of a common *hmox1* signature in response to PTM may be useful for further research, the consequences on tumor metastasis in vivo are controversial. In breast cancer cells, we found a strong increase in *hmox1* levels after PTM exposure, and such an upregulation was shown to be associated with a significant reduction of invasive properties in MCF7 and MDA-MB-231 cells [50]. Similar observations were made with colon cancer cells [51,52]. As a mechanism, the inhibition of matrix-metalloproteinase (MMPs) through HO1-derived CO was proposed, as MMPs facilitate the degradation of extracellular matrix needed for metastatic spread. However, several reports argue for a role of HO1 in promoting metastatic spread in cancer [53,54,55]. This is linked especially to its constitutive upregulation in many types of tumors as well as studies showing that *hmox1* downregulation is associated with better outcome. Specifically, among non-responders to chemotherapy in a cohort of head and neck cancer patients, *hmox1* was the most upregulated gene that is identified in a transcriptomic microarray study as compared to complete responders [56].

*Hmox1* expression is tightly linked to regulation of immunity and inflammation [39]. Investigating a panel of well-recognized markers for immunogenic cell death (ICD) that are involved in immunological recognition and removal of tumor cells [41,57,58], we identified only heat-shock protein 90 (HSP90) to be upregulated in SK-Mel 28 cells. However, the profound upregulation of HSP90 on SK-Mel 28 cell membrane in response to low-dose oxidative stress may be a valuable research target for tumor immunology. HSP90 is a damage-associated molecular pattern (DAMP) that can facilitate the uptake of tumor cells by dendritic cells to induce antitumor immune responses [59]. Release of DAMPs and enhanced expression of ICD-relevant markers has been shown for plasma-derived ROS/RNS in various type of tumor cells [60,61,62]. Yet, our PTM was rather tumor-static than tumor-toxic, possibly contributing to the lack modulation with all except one marker in almost all cell lines. With respect to the three cell lines (HT29, SW480, MCF7) with the strongest increase in *hmox1* levels, a number of chemokines/cytokines/growth factors were all increased in tendency in response to PTM, namely CCL17, IFNγ, IL1β, IL6, IL8, IL10, IL12, TGFβ, TNFα, and VEGF. A correlation between increase of *hmox1* and IL8 as well as VEGF was reported before being major angiogenic drivers in tissues [63]. In a murine carcinogenesis model, IL1β models for only non-significantly increased with a significant increase in *hmox1* [64], corroborating our findings. HO1 is also described to increase IL10 production [65], as seen in our subset of cell with high *hmox1* expression. In general, this subsets had a consistent increase in both pro-inflammatory (CCL17, CCL17, IFNγ, IL1β, IL6, IL12, and TNFα) and anti-inflammatory (IL8, IL10, TGFβ, and VEGF) cytokines, underlining the complex effects that HO1 has on inflammation.

The effect-inducing agent in our study was cell culture medium exposed to cold physical plasma, a partially ionized gas operated at room temperature. A number of reactive oxygen and nitrogen species were so far identified in plasma-treated liquids [66], among them e.g., superoxide anion, singlet oxygen, nitrite, nitrate, peroxynitrite, hydrogen peroxide, hydroxyl radical, nitric oxide, ozone, and atomic oxygen [67,68,69,70,71]. However, most of these species quickly deteriorate to some major long-lived oxidants, which are likely to mediate the main effect that was observed with PTM. Only recently, reports dealt with post-translational modifications on biomolecules and proteins that are present in liquid media [72], which may be recognized by cells and their oxidation-sensitive receptor repertoire. Oxidative stress is sensed through the *nrf2*/Keap1 pathway [73], which leads to the activation of antioxidative responses elements (ARE), including transcriptional targets, such as *vegf*, *hmox1*, *bach1*, *fos*, *junb/d*, *maf*, and others [74]. While we here identified a consistent upregulation of *hmox1* in eight tumor cell types following exposure to PTM, no changes in *nrf2* levels were observed. This is consistent with a previous report in human keratinocytes were *nrf2* mRNA levels peaked at 20 min following exposure to PTM, whereas that of *hmox1* peaked at 3 h [75]. 

## 5. Conclusions

The main aim of this study was to identify a common redox-related transcriptional signature in eight human cancer lines following exposure to physical plasma-treated medium (PTM). We not only identified transcriptional levels of *hmox1* as mutual response element but also provided useful tools, such as a 22 target transcriptional map and cytokine/chemokine patterns in all cell lines to further study the role of other redox proteins that signal with plasma-derived ROS/RNS.

## Figures and Tables

**Table 1:** Immunogenic cancer cell death (ICD)-related surface molecules investigated in this study.

Abbreviation	Full Name	Physiological Relevance
CD47	Cluster of differentiation 47	Serves as “don’t eat” me signal by binding CD172a on myeloid cells to prevent phagocytosis of malignant and non-malignant cells [34]
CRT	Calreticulin	ICD marker and serves as “eat-me” signal on non-malignant and malignant cells to promote phagocytosis by myeloid cells [35]
HLA-ABC	Human leukocyte antigen A, B, and C	Major histocompatibility complex I (MHC class I), serves to present peptides of endogenous protein to patrolling CD8^+^ T-cells; important for antitumor immune responses [36]
HSP70	Heat-shock protein 70	ICD marker, chaperone, and damage-associated molecular pattern (DAMP) [37]
HSP90	Heat-shock protein 90	ICD marker, chaperone, and damage-associated molecular pattern (DAMP) [38]
